# Modulation of Carnitine Palmitoyl Transferase 1b Expression and Activity in Muscle Pathophysiology in Osteoarthritis and Osteoporosis

**DOI:** 10.3390/biom14101289

**Published:** 2024-10-12

**Authors:** Chiara Greggi, Manuela Montanaro, Maria Giovanna Scioli, Martina Puzzuoli, Sonia Gino Grillo, Manuel Scimeca, Alessandro Mauriello, Augusto Orlandi, Elena Gasbarra, Riccardo Iundusi, Sabina Pucci, Umberto Tarantino

**Affiliations:** 1 Department of Clinical Sciences and Translational Medicine, University of Rome Tor Vergata, Via Montpellier 1, 00133 Rome, Italy; chiara.greggi@gmail.com (C.G.); elenagasbarra@tiscali.it (E.G.); riccardo.iundusi@uniroma2.it (R.I.); umberto.tarantino@uniroma2.it (U.T.); 2 Department of Biomedicine and Prevention, University of Rome Tor Vergata, Via Montpellier 1, 00133 Rome, Italy; manuelamontanaro1991@gmail.com (M.M.); martina.puzzuoli@gmail.com (M.P.); orlandi@uniroma2.it (A.O.); sabinapuc@yahoo.it (S.P.); 3 Department of Orthopaedics and Traumatology, “Policlinico Tor Vergata” Foundation, Viale Oxford 81, 00133 Rome, Italy; s.ginogrillo@gmail.com; 4 Department of Experimental Medicine, University of Rome Tor Vergata, Via Montpellier 1, 00133 Rome, Italy; manuel.scimeca@uniroma2.it (M.S.); alessandro.mauriello@uniroma2.it (A.M.); 5 Faculty of Medicine and Surgery, University “Our Lady of Good Counsel”, Rruga Dritan Hoxha, 1000 Tirana, Albania

**Keywords:** osteoporosis, osteoarthritis, muscle atrophy, energy metabolism, oxidative stress, mitochondria biogenesis, myoblast regeneration

## Abstract

In the pathophysiology of osteoarthritis and osteoporosis, articular cartilage and bone represent the target tissues, respectively, but muscle is also involved. Since many changes in energy metabolism occur in muscle with aging, the aim of the present work was to investigate the involvement of carnitine palmitoyl transferase 1b (Cpt1b) in the muscle pathophysiology of the two diseases. Healthy subjects (CTR, *n* = 5), osteoarthritic (OA, *n* = 10), and osteoporotic (OP, *n* = 10) patients were enrolled. Gene expression analysis conducted on muscle and myoblasts showed up-regulation of *CPT1B* in OA patients; this result was confirmed by immunohistochemical and immunofluorescence analyses and enzyme activity assay, which showed increased Cpt1b activity in OA muscle. In addition, CPT1B expression resulted down-regulated in cultured OP myoblasts. Given the potential involvement of Cpt1b in the modulation of oxidative stress, we investigated ROS levels, which were found to be lower in OA myoblasts, and gene expression of nicotinamide adenine dinucleotide phosphate hydrogen oxidase 4 (Nox4), which resulted up-regulated in OA cells. Finally, the immunofluorescence of BCL2/adenovirus E1B 19 kDa protein-interacting protein 3 (Bnip3) showed a decreased expression in OP myoblasts, with respect to CTR and OA. Contextually, through an ultrastructural analysis conducted by Transmission Electron Microscopy (TEM), the presence of aberrant mitochondria was observed in OP muscle. This study highlights the potential role of Cpt1b in the regulation of muscle homeostasis in both osteoarthritis and osteoporosis, allowing for the expansion of the current knowledge of what are the molecular biological pathways involved in the regulation of muscle physiology in both diseases.

## 1. Introduction

Osteoporosis and osteoarthritis represent two age-related diseases of the musculoskeletal system characterized by a strong global impact [1,2]. Osteoporosis is considered one of the most critical health problems after cardiovascular disease: it is characterized by an alteration in the microarchitecture of bone tissue and a decrease in bone mineral density (BMD), which are reflected in a decrease in the strength of the bone itself, leading to an increased risk of low-energy fractures [3,4]. Osteoarthritis represents a chronic degenerative and debilitating condition affecting the entire joint and is characterized by hyaline articular cartilage damage, tissue hypertrophy, subchondral bone degradation, tendon and ligament instability, and synovial hypervascularization. This condition is common in older adults, causing progressive walking disability, resulting in reduced activities of daily living [5,6]. In the pathophysiology of these diseases, the target is not only the bone tissue but also the muscle: in fact, bone and muscle represent a functional unit in which the two tissues are intimately connected to each other, both anatomically and mechanically. Consequently, alterations in the physiology of bone tissue lead to consequences on the structure and function of muscle and vice versa [7]. In fact, during aging, bone tissue deterioration is generally accompanied by a concomitant and progressive loss of muscle mass, strength, and function, known as sarcopenia. This condition leads to an increased risk of falls and fragility fractures, morbidity, and an overall worsening of quality of life [8]. Factors leading to the onset of sarcopenia are prolonged periods of physical inactivity, eating disorders and malnutrition, and chronic diseases that have deleterious effects on muscle mass and function [9,10]. There are numerous age-related hormonal and metabolic changes that greatly contribute to the decline of numerous physiological functions, as occurs in sarcopenia. The decline in hormone production is, in fact, associated with an increase in fat mass, a decrease in lean mass, and with a reduction in the number and functionality of mitochondria in the muscle that contributes to a reduction in contractile force generation [11,12]. Indeed, altered mitochondrial activity leads to a reduction in adenosine triphosphate (ATP) production, a decrease in mitochondrial protein synthesis that ultimately results in the onset of a state of muscle atrophy [13]. In addition, a state of chronic low-grade inflammation and oxidative stress represent the common denominator in osteoarthritis and osteoporosis. Indeed, during aging, cartilage and bone cells are exposed to various pathophysiological mediators, including reactive oxygen species (ROS) and numerous pro-inflammatory cytokines. Therefore, the resulting damage to the cellular microenvironment triggers cellular stress that contributes to the development of these diseases while also compromising the regenerative processes that serve to counteract and mitigate the degeneration of affected target tissues [14].

Carnitine palmitoyl transferase 1 (Cpt1) is a protein that plays a primary role in cellular energy metabolism: it resides in the outer mitochondrial membrane and is involved in the transport of long-chain fatty acids into the mitochondria for β-oxidation and subsequent ATP synthesis [15]. Three different protein variants belonging to the Cpt1 family have been identified: Cpt1a (also called L-Cpt1), Cpt1b (or M-Cpt1), and the most recently described variant, Cpt1c. Cpt1a appears to be most localized in the liver, pancreas, kidney, brain, blood, and embryonic tissues. Cpt1c is mainly expressed in the endoplasmic reticulum of neurons, while Cpt1b is the only protein belonging to this family that shows purely muscular expression as well as being expressed in brown adipose tissue and the heart [16].

The identification of the molecular and cellular determinants involved in the pathogenesis of osteoarthritis and osteoporosis may open new perspectives for the development of therapeutic strategies that can prevent/treat these diseases. To this end, it is necessary to expand the current knowledge of the pathophysiological mechanisms underlying the onset of these diseases, focusing not only on bone and cartilage but also on muscle as it is closely connected to bone. Therefore, the purpose of this study was to preliminarily investigate the potential involvement of the muscle protein Cpt1b in the pathophysiology of osteoarthritis and osteoporosis by evaluating the expression and activity of the enzyme in the muscle tissue and myoblasts of healthy (CTR), osteoporotic (OP), and osteoarthritic (OA) subjects. In addition, since the literature suggests that Cpt1 family proteins may be involved in the oxidative stress response, we evaluated this in muscle tissue, since it is generally analyzed in the two main target tissues of osteoporosis and osteoarthritis, namely, bone and cartilage, respectively. Finally, given the mitochondrial localization of the enzyme, we assessed the quality of mitochondria and investigated levels of mitophagy in muscle tissue.

## 2. Materials and Methods

### 2.1. Subjects

The study was approved by the Ethical Board of “Policlinico Tor Vergata” (approval reference number #17/21; approval date 1 March 2021). Informed consent was obtained from all the participants and all experimental procedures were carried out according to The Code of Ethics of the World Medical Association (Declaration of Helsinki). Subjects were divided into three groups of analysis: 10 osteoporotic patients (OP) who underwent surgery for fragility fractures following low-energy trauma, 10 osteoarthritic patients (OA) who underwent surgery for osteoarthritis of the hip, and 5 healthy controls (CTR) who underwent surgery for high-energy fractures. Individuals affected by malignancies, endocrine disorders affecting bone and mineral metabolism, autoimmune diseases, and bone disorders other than primary osteoporosis were excluded from the study as well as those who underwent long-term therapy with drugs interfering with bone metabolism, sex hormone replacement therapy, and/or antifracture and/or osteoanabolic therapies.

### 2.2. Clinical and Biochemical Parameters

Densitometric diagnosis of OP based on Dual-energy X-ray absorptiometry (DXA) evaluation of mineral density was carried out on each subject with a Lunar DXA apparatus (GE Healthcare, Madison, WI, USA). Lumbar spine (L1–L4) and femoral (neck and total) scans were performed according to the manufacturer’s recommendation [17]. The unit of measurement is represented by SD from the mean bone mass peak (*t*-score), and BMD was measured (in grams per square centimeter), with a coefficient of variation of 0.7%, on the uninjured limb. A *t*-score value ≥ −1 indicates a normal condition, a *t*-score between −1 and −2.5 indicates osteopenia, and a *t*-score < −2.5 indicates a condition of osteoporosis. For OA patients, measurements were performed on the non-dominant side, with the participants supine on an examination table with their limbs slightly abducted. DXA exam was performed 1 day before surgery for OA patients and 1 month after surgery for OP patients. Hip X-rays were performed in order to check the fracture or to assess hip OA. The Kellgren-Lawrence scale was used in order to determine the severity of OA [18]. Two orthopedists independently assessed all radiographs. Patients with a grade of K−L ≥ 2 were considered osteoarthritic. Calcium, PTH, and 25-(OH)-VitD levels were measured in fasting venous blood samples. Finally, to assess muscle quality status, a histomorphometric analysis of muscle tissue was conducted, measuring the diameter of muscle fibers, and an immunohistochemical analysis of fast myosin was conducted to calculate the percentage of type II muscle fibers (see below). The detailed clinical characteristics of the study subjects are summarized in Table 1.

### 2.3. Samples’ Collection

*Vastus lateralis* muscle biopsies were collected during hip arthroplasty surgeries in OP and OA patients and during osteosynthesis surgeries for CTR patients. The specimens were stored immediately in complete medium for subsequent setup of primary myoblast cultures (see below), preserved in 4% paraformaldehyde for 24 h, and, finally, paraffin embedded for subsequent histological analysis.

### 2.4. Human Myoblasts’ Primary Cell Culture

Muscle tissue samples taken during surgery were first washed in phosphate-buffered saline (PBS) and shredded into small pieces with the help of scissors. The tissue pieces were transferred to a 15 mL Falcon with PBS and centrifuged at 360 rcf for 30 s. Then, after removing the supernatant, the tissue was subjected to digestion in a 2.5 mg/mL solution of Collagenase NB 4G Proved grade 0.18 U/mg (SERVA Electrophoresis GmbH, Heidelberg, Germany) diluted in Dulbecco’s modified Eagle medium (DMEM) F12 medium (MS01801009, Biowest, Nuaillé, France) in oscillation, at 37 °C for 1 h. After that time, centrifugation at 360 rcf for 10 min was performed. After that, the supernatant was removed and the digestion product was resuspended in complete growth medium: Ham’s F14 (L0138-500, Biowest, Nuaillé, France) supplied with 15% FBS (F7524, Merck, Darmstadt, Germany) insulin 1 mg/mL (I9278, Merck), 2 mM L-glutamine (G7513, Merck), 100 U/mL penicillin-100 µg/mL streptomycin (P4333, Merck) and 0.25 µg/mL Amphotericin B (A2942, Merck), FGF 5 µg/mL (RP-8628, Thermo Fisher Scientific, Waltham, MA, USA), and EGF 10 µg/mL (01-AA060-0010, ISOkine, ORF Genetics, Kópavogur, Iceland). At this point, the cell suspension was filtered first through 70 µm filter and then through 40 µm filter and was centrifuged at 360 rcf for 10 min. After that, the cell pellet was resuspended in complete culture medium and the cells were left in culture until 80% confluence was reached. When the cell number reached the minimum amount of about 5 million, specific selection of myoblasts by specific antibody for CD56 detection was carried out (EasySep™ Human CD56 Positive Selection Kit II, Stemcell Technologies, Vancouver, BC, Canada). Myoblasts were characterized for Myoblast determination protein 1 (MyoD) expression at passage 1 (after the explant passage 0). Then, a second characterization was performed in parallel to the seeding for the experiments (strictly at passage 3–4). For myotubes, differentiation was induced at 90% of confluence of myoblast cultures, strictly at passage 3–4, by adding culture medium without growth factors with only human insulin, for 15 days. Differentiation was continuously monitored by light microscope and confirmed by myosin heavy chain 1 (MYH1) up-regulation. To perform immunocytochemistry analysis and enzyme activity assay of CPT1B, 5000 cells per well were plated in 24-well multiwells; for the purpose of protein extraction, 50,000 cells were plated in 60 mm Petri dishes.

### 2.5. RNA Extraction and qRT-PCR Analysis

Total RNA extraction was performed by manual extraction. Chloroform totaling 0.1 mL was added to 0.5 mL of TRIzol reagent (Thermo Fisher Scientific, Waltham, MA, USA), shaken vigorously for 15 s, and incubated at room temperature for 3 min. Samples were centrifuged at 12,000× *g* for 12 min at 4 °C. After collection, the upper phase was mixed with isopropyl alcohol and centrifuged at 12,000× *g* for 8 min at 4 °C. The sediment was washed with 75% ethanol and air-dried for approximately 30 min. Purified RNA was dissolved in 30 μL of RNase-free water and stored at 80 °C. About 500 ng of RNA was purified and subjected to reverse transcription using the cDNA with High-Capacity Reverse Transcription kit (Thermo Fisher Scientific, Waltham, MA, USA). Real-time PCR was performed on an Applied Biosystems^®^ 7500 Fast Real-Time PCR System (Life Technologies; Carlsbad, CA, USA). The qPCR analysis was conducted using a Power SYBR green kit (Thermo Fisher Scientific, Waltham, MA, USA) and the following cycles: 95 °C for 10 min, followed by 95 °C for 15 s and 60 °C for 1 min for 40 cycles. The sequences of primers are reported in Table 2. The relative difference in CPT1B and NOX4 gene expression among OP, OA, and CTR subjects was calculated using 2 CT method and normalized to *GAPDH* as the internal control.

### 2.6. Reactive Oxygen Species (ROS) Quantification Assay

ROS level in myoblasts were measured by 5-(and-6)-chloromethyl-2′,7′-dichlorodihydrofluorescein diacetate, acetyl ester (CM-H2DCFDA) fluorescence method (C6827, Thermo Fisher Scientific) [19]. Briefly, cells were seeded in 96-well chambers at a density of 8000 cell/cm^2^ with 100 µL of complete culture medium and were incubated overnight. The next day, dichlorofluorescein fluorescence was measured by a fluorescence microtiter plate reader (Glomax Explorer, Promega, Milan, Italy). Results were expressed as the mean of three independent experiments performed in triplicate.

### 2.7. Enzyme Activity Assay of Cpt1b

Total protein extract was used to evaluate the enzymatic activity of Cpt1b: 850 μL of DNTB buffer (Tris-HCl 1.5 M, EDTA 0.5 M, DNTB 2 mM) were incubated with 40 μg of protein extracts at room temperature for 20 min. Absorbance was measured at 412 nm. Then, 50 μL of Palmitoyl-CoA 1 mM ± 5 μL of L-Carnitine 1 mM were added; after mixing, absorbance was measured at 412 nm. Incubation at 37 °C was performed and the reading was repeated.

### 2.8. Histomorphometric and Immunohistochemical Analyses

Muscle biopsies were fixed in 4% paraformaldehyde for 24 h and paraffin embedded. Then, 3 μm-thick sections were stained using hematoxylin and eosin (H&E) (05-06002, Bio-Optica, Milan, Italy) to perform histomorphometric analysis. Ten microscopic images, randomly selected, were evaluated for each biopsy sample. Images were acquired at 20× magnification using a Nikon upright microscope ECLIPSE CiS/(Nikon Corporation, Tokyo, Japan) connected to a Nikon digital camera. Image analysis was performed using NIS-Elements software (5.30.01; Laboratory Imaging, Prague, Czech Republic), according to the manufacturer’s instructions. For histomorphometric analysis, the diameter of 200 fibers per section was measured. For immunohistochemical analysis, 3 μm-thick muscle sections were incubated with polyclonal rabbit anti-Cpt1b antibody (Thermo Fisher Scientific, Waltham, MA, USA), monoclonal mouse anti-BCL2/adenovirus E1B 19 kDa protein-interacting protein 3 (Bnip3) antibody (Thermo Fisher Scientific, Waltham, MA, USA), and monoclonal mouse anti-Fast Myosin Skeletal Heavy chain antibody [MY-32] (ab51263, Abcam, Cambridge, UK). Washing was performed with PBS/Tween20 pH 7.6 (UCS Diagnostic, Rome, Italy); reactions were revealed by horseradish peroxidase (HRP)-3,3diaminobenzidine (DAB) Detection Kit (UCS Diagnostic, Rome, Italy). Immunohistochemical positivity was assessed on digital images (see above): for each section, 10 fields at 20× magnification were analyzed.

### 2.9. Immunofluorescence Analysis

Immunofluorescence first required fixation with paraformaldehyde. Next, the cells were permeabilized with a solution of PBS ± 0.2% Tween (Thermo Fisher Scientific) for 5 min. Following washing with 1X PBS, the cells were incubated for 1 h with the primary antibody Cpt1b (Thermo Fisher Scientific, Waltham, MA, USA) and Bnip3 (Thermo Fisher Scientific, Waltham, MA, USA). After a further wash in PBS 1X, the cells were incubated with the secondary antibody; a further wash was performed, incubation with Hoechst (1:1500, Merck, Darmstadt, Germany) for 45 min was performed, and, finally, a final wash in PBS 1X was performed. Images were captured with confocal microscope through the use of NIS-Elements software (5.30.01; Laboratory Imaging, Prague, Czech Republic).

### 2.10. Transmission Electron Microscopy (TEM)

From muscle tissue biopsies, 1 mm^3^ of each sample was fixed in 4% paraformaldehyde for 24 h. After fixation, washings with 0.1 M phosphate buffer were carried out and tissues were post-fixed in 2% osmium tetroxide. Samples were dehydrated in 30%, 50%, 70%, and 95% ethanol scale. For EPON embedding, dehydration proceeded to absolute ethanol and propylene oxide; then, samples were embedded in resin (Agar Scientific, Stansted Essex CM24 8GF UK). Seventy nm ultra-thin sections were cut, mounted on copper grids, and examined with TEM (Model JEM1400, JEOL USA, Inc., 11 Dearborn Road, Peabody, MA 01960, USA; Digital Micrograph TM Software version 702.107).

### 2.11. Statistical Analysis

Data were analyzed with GraphPad Prism 5.0 (GraphPad Software, Inc., La Jolla, CA, USA). Before using statistical test procedures, the assumptions of normality were verified for each variable, applying the D’Agostino and Pearson normality test. A non-parametric Kruskal-Wallis test was used for variables showing a skewed distribution, whereas data following a normal distribution were processed with Welch’s test. Differences were considered significant when the *p* value was <0.05 (* *p* < 0.05, ** *p* < 0.01, *** *p* < 0.001). 

## 3. Results

### 3.1. Clinical Characteristics of Individuals Included in the Study

The baseline characteristics of the study population (OP: *n* = 10; OA: *n* = 10; CTR: *n* = 5) are reported in Table 1. The OP and OA patients were younger than CTR patients (respectively, 64.3 ± 4.8 and 66.6 ± 6.2 vs. 30.0 ± 6.9; *p* < 0.001), while the age difference between OP and OA was not statistically significant. The assessment of the bone mineral density of the lumbar spine, total femur, and femoral neck, expressed as BMD and *t*-score values, showed a statistically significant difference between CTR subjects and OP patients (*p* < 0.001) and between OA and OP patients (*p* < 0.001): OP patients were, in fact, characterized by lower BMD and *t*-score values at the lumbar spine, femoral neck, and whole femur, with respect to CTR and OA. No significant differences between CTR and OA were observed. In addition, there was no statistically significant difference in the circulating markers of Calcium, 25-(OH)-VitD, and PTH among any of the groups.

### 3.2. Muscle Quality Is Comparable between CTR and OA but Is Lower in OP Patients

**Figure 1:**
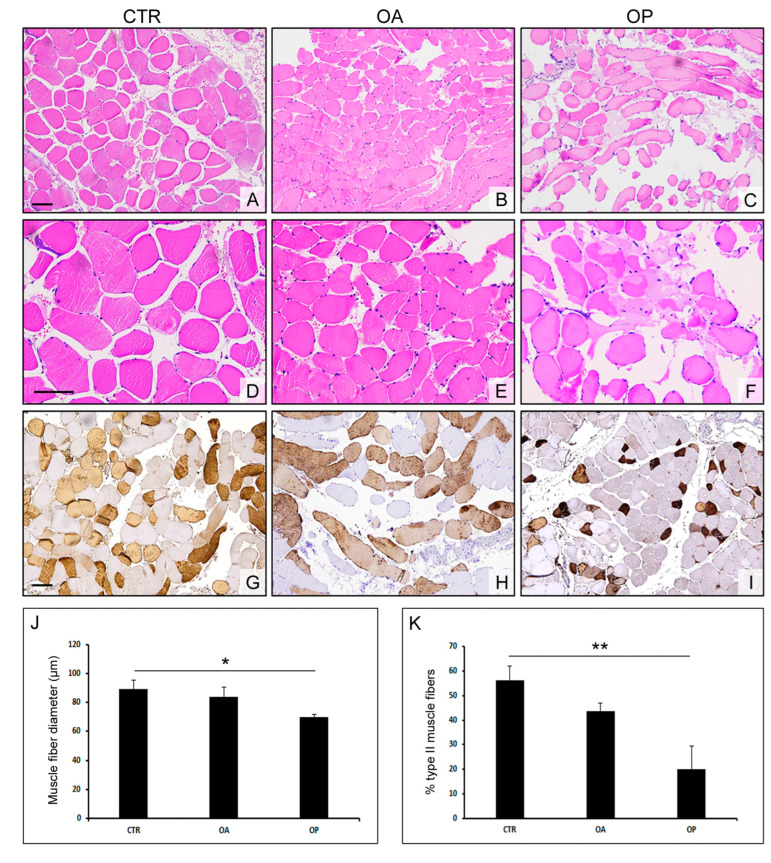
**Morphological analyses and characterization of muscle fibers from CTR, OA, and OP patients.** (**A**–**F**) Representative images at different magnifications of hematoxylin–eosin-stained muscle sections from healthy (CTR), OA, and OP patients (scale bar = 100 µm). (**G**–**I**) Immunohistochemical staining for myosin fast (scale bar = 100 µm). (**J**,**K**) Graphs showing morphometric evaluation of muscle fibers’ diameter and the percentage of type II muscle fibers in CTR, OA, and OP patients. Results are reported as mean ± SEM; *t*-test: * *p* < 0.05; ** *p* < 0.01.

Histomorphometric analyses of all the groups’ biopsies were performed to assess the diameter of the muscle fibers. These analyses showed that the muscle tissue of CTR subjects and OA patients was comparable while the diameter of the muscle fibers of OP group patients was smaller than that observed in the CTR group (*p* < 0.05). In contrast, the difference observed between OA and OP muscle fibers was not statistically significant. To further characterize the quality of the muscle tissue of the three patient groups, we next performed immunohistochemical analyses of fast myosin expression to identify type II muscle fibers. This investigation showed a higher percentage of fibers in CTR subjects than in OA patients but not significantly so, while the amount of type II fibers was significantly higher in healthy subjects than in OP patients, characterized by a higher degree of muscle atrophy (*p* < 0.05). In contrast, the difference was not statistically significant between the OA and OP groups (Figure 1).

### 3.3. The Expression and Activity of CPT1B Is Increased in OA Muscle

**Figure 2:**
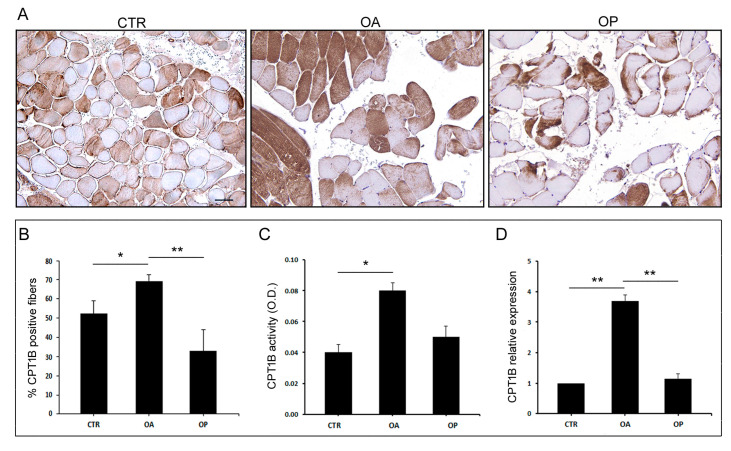
**CPT1B expression and activity in muscle tissue from CTR, OA, and OP patients.** (**A**) Immunohistochemical staining for Cpt1b of muscle sections from healthy (CTR), OA, and OP patients (scale bar = 100 µm). (**B**) Graph showing the percentage of CPT1B-positive fibers in CTR, OA, and OP patients. (**C**) Cpt1b activity in muscle tissues from CTR, OA, and OP patients. (**D**) *CPT1B* transcript levels in muscle tissues from CTR, OA, and OP patients. Results are reported as mean ± SEM; *t*-test: * *p* < 0.05; ** *p* < 0.01. Abbreviation: O.D., optical density.

To investigate the potential involvement of Cpt1b in the regulation of muscle energy metabolism, we first investigated the amount of Cpt1b-positive muscle fibers, by immunohistochemical analysis. The percentage of Cpt1b-positive fibers was 69.4% in OA patients compared with 52.3% in healthy subjects (*p* < 0.05); however, 32.8% positive fibers were observed in the muscle of OP patients, significantly lower than in OA patients (*p* < 0.01) but not significantly lower than in CTR subjects. An analysis of the enzymatic activity of Cpt1b was also carried out, using protein extracts from the muscle tissue of the three patient groups. This investigation showed a significant increase in Cpt1b activity in the OA group, compared to healthy subjects (*p* < 0.05). The increase was not statistically significant between CTR and OP subjects and between OA and OP groups. Finally, the gene expression level of the enzyme in the muscle tissue of the three patient groups was analyzed. Gene expression analysis showed a significant increase in the expression of *CPT1B* in the muscle of OA patients compared with both healthy subjects (*p* < 0.01) and OP patients (*p* < 0.01). In both cases, an almost 4-fold increase in expression was observed. No significant difference in expression was observed between CTR subjects and OP patients (Figure 2).

### 3.4. The Expression of CPT1B Is Increased in OA Cultured Myoblasts

**Figure 3:**
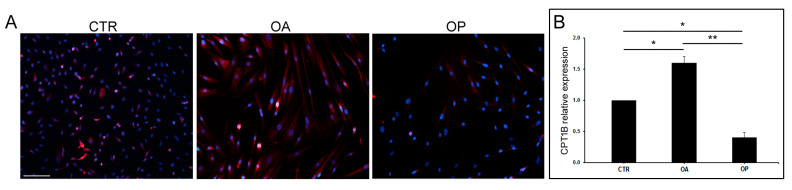
**CPT1B expression in cultured myoblasts from CTR, OA, and OP patients.** (**A**) Immunofluorescence for CPT1B in cultured myoblasts from healthy (CTR), OA, and OP patients (scale bar = 100 µm). (**B**) *CPT1B* transcript levels in cultured myoblasts from CTR, OA, and OP patients. Results are reported as mean ± SEM; *t*-test: * *p* < 0.05, ** *p* < 0.01.

An immunofluorescence analysis was first conducted, which showed that there was a different morphology of the cultured myoblasts from the three groups of patients; specifically, OA and OP myoblasts appeared larger and different in size than CTR (images shown were obtained at the same magnification). In addition, a higher expression of Cpt1b was observed in OA cells, confirming the results previously obtained by immunohistochemical analysis. Subsequently, gene expression analysis was conducted, the results of which confirmed what was obtained on muscle tissue: the expression of *CPT1B* was, in fact, higher in OA patients both compared to healthy subjects (*p* < 0.05) and compared to OP patients (*p* < 0.01). The expression of the enzyme was also significantly increased in CTR subjects compared to OP patients (*p* < 0.05) (Figure 3).

### 3.5. Oxidative Stress Levels Are Lower in OA Muscle than in OP

**Figure 4:**
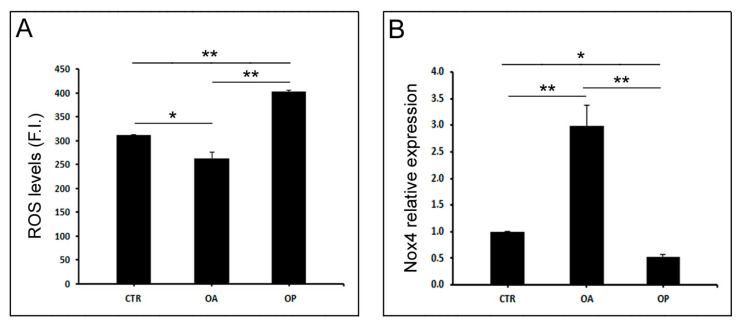
**Oxidative stress and *NOX4* expression in cultured myoblasts from CTR, OA, and OP patients.** (**A**) ROS levels in cultured myoblasts from healthy (CTR), OA, and OP patients. (**B**) *NOX4* transcript levels in cultured myoblasts from CTR, OA, and OP patients. Results are reported as mean ± SEM; *t*-test: * *p* < 0.05; ** *p* < 0.01. Abbreviation: F.I., fluorescence intensity.

Since the literature suggests that Cpt1 family proteins may be involved in the oxidative stress response, we carried out a ROS quantification assay in myoblasts isolated from muscle tissue to quantify the level of oxidative stress in the muscle of the three groups of patients. The intracellular ROS quantification assay showed a lower level of ROS in myoblasts isolated from the muscle of OA patients, both compared with CTR subjects (*p* < 0.05) and OP patients (*p* < 0.01). In contrast, the level of ROS was higher in OP myoblasts than in CTR subjects (*p* < 0.01). Subsequently, we investigated the gene expression levels of *NOX4* in myoblasts isolated from the muscle tissue of CTR, OA, and OP patients. This analysis showed that *NOX4* expression was higher in OA myoblasts compared with both CTR subjects (*p* < 0.01) and OP myoblasts (*p* < 0.01), whereas gene expression was down-regulated in OP myoblasts compared with both CTR subjects (*p* < 0.05) (Figure 4).

### 3.6. Bnip3 Has a More Localized Expression in OA Myoblasts

**Figure 5:**
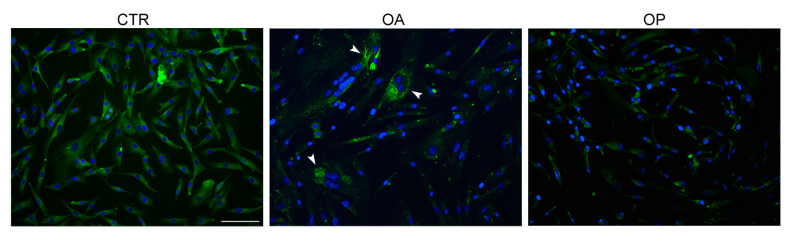
**Bnip3 localization in cultured myoblasts from CTR, OA, and OP patients.** Immunofluorescence for Bnip3 in cultured myoblasts from CTR, OA, and OP patients (scale bar = 100 µm). Specifically, in CTR myoblasts, the protein is diffuse along all the cytoplasm, whereas in OA myoblasts, Bnip3 is aggregated in numerous and distinct spots (puncta) similar to autophagosomes (white arrowheads). OP myoblasts revealed a lower expression of Bnip3 with no evident puncta.

Given the mitochondrial localization of Cpt1b, we conducted a qualitative analysis of the expression of Bnip3, which plays a primary role in the mitophagy process, to broaden our understanding of the pathogenic mechanisms in which Cpt1b appears to be involved. Therefore, we performed an immunofluorescence analysis on myoblasts isolated from the muscle tissue of the three patient groups; this investigation revealed a different expression pattern among CTR subjects, OA and OP patients. Specifically, Bnip3 expression was higher in CTR myoblasts compared with OA and OP myoblasts. In healthy myoblasts, Bnip3 expression appeared to be homogeneously diffused along all the cytoplasm. Interestingly, on the other hand, a peculiar distribution of the protein was observed in OA myoblasts, which appeared to be localized in small and numerous spots (puncta) within the cytoplasm, similar to autophagosomes. Finally, in OP myoblasts, the expression of BNIP3 was lower and the distribution quite widespread compared with both CTR and OA myoblasts (Figure 5).

### 3.7. Ultrastructural TEM Analysis Revealed the Presence of Aberrant Mitochondria in the Muscle of OP Patients

**Figure 6:**
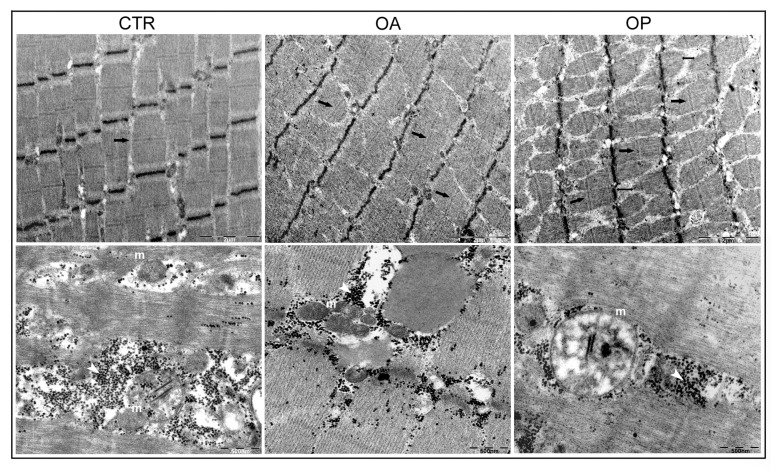
**Ultrastructural analysis of muscle tissue from CTR, OA, and OP patients.** Transmission electron microscopy (TEM) of muscle tissue from CTR, OA, and OP patients. Images of CTR muscle fibers show the presence of well-organized sarcomeres (arrows), as well as for OA patients, while sarcomeres of OP muscle appear not well defined. Numerous glycogen granules (white arrowheads) characterize the muscle fibers of CTR and OA patients in contrast with OP group. Increased intermyofibrillar space is also observed in OP muscle, in which their presence appears reduced (double arrow). In addition, mitochondria (m) in the muscle tissue of CTR and OA patients display intact and well-organized cristae; in the muscle tissue of OP patients, on the contrary, mitochondria appeared irregularly shaped with loose mitochondrial cristae.

To perform a more in-depth qualitative tissue analysis, muscle tissue from the three groups of patients was processed to perform TEM ultrastructural evaluation. Firstly, this analysis revealed the presence of well-organized sarcomeres only in CTR subjects and OA patients, while they appeared not well-organized in the muscle tissue of the OP group, confirming the worse muscle qualitative state characterizing these patients. In addition, the muscle of CTR and OA patients was found to be characterized by the presence of numerous glycogen granules, whose presence appeared reduced in the OP muscle. In addition, we observed an increased intermyofibrillar space in the OP group tissue, which is typical of atrophic muscle tissue. Finally, in the muscle tissue of CTR and OA patients, the presence of mitochondria (m) characterized by intact and well-organized mitochondrial cristae was observed; in the muscle tissue of OP patients, on the contrary, mitochondria appeared irregularly shaped and also seemed to have lost the integrity of mitochondrial cristae. The presence of numerous small vesicular structures accumulated in the cytoplasm was also noted (Figure 6).

## 4. Discussion

Osteoarthritis and osteoporosis represent the most common age-related diseases affecting the musculoskeletal system. In both diseases, an important target is bone tissue: in osteoporosis, there is a reduction in BMD resulting in an increased risk of low-energy fractures, while the degeneration of articular cartilage seen in osteoarthritis results in inevitable damage to subchondral bone as well [3,4]. Not very often, when referring to the pathophysiology of both diseases, reference is made to the involvement of muscle tissue. In fact, bone and muscle are intimately connected to each other, both biomechanically and biochemically: bone provides insertion sites for skeletal muscle through the bone–muscle junction, while contraction of skeletal muscle drives movement of the lever system bone. Furthermore, it is known that there are factors secreted by bone and skeletal muscle that mediate the interaction between the two tissues [20]. Generally, depending on the severity of the reduction in BMD and degree of joint function, both osteoporosis and osteoarthritis may be accompanied by a concomitant decrease in muscle mass and strength, which is referred to as sarcopenia [21]. In fact, the term sarcopenic osteoarthritis and osteosarcopenia has been coined to describe the concomitant presence in the same individual of sarcopenia and osteoarthritis, or sarcopenia and osteoporosis, respectively [22,23]. If the state of sarcopenia is particularly severe, sufferers are forced into a sedentary lifestyle, which can also lead to disability and a consequent increase in mortality [7].

During aging, both structural and metabolic profound changes occur in muscle. At molecular and biological levels, this condition is indeed characterized by an increased protein degradation, apoptosis, altered autophagy process, altered myogenesis, and mitochondrial dysfunctions. The latter play a particularly relevant role, as they result in changes in energy metabolism, impairing the contractile capacity of the muscle fiberand triggering an inevitable process of muscle atrophy [24].

The aim of the present work was, therefore, to analyze the expression and function of the muscle protein Cpt1b, an enzyme located in the outer mitochondrial membrane involved in the import of long-chain fatty acids into the mitochondria for β-oxidation. These investigations were conducted on muscle tissue from CTR, OA, and OP subjects and on myoblasts isolated from the same tissues of the three patient groups.

First, the muscle quality of the three experimental groups was analyzed by assessing the diameter of muscle fibers and the percentage of type II fibers. In agreement with the literature, muscle quality was found to be comparable between healthy subjects and OA patients, given the larger diameter and percentage of type II fibers observed in these patients, compared with the muscle tissue of the OP group, which was characterized by smaller-diameter fibers and a lower percentage of fast myosin-positive muscle fibers [25,26].

Next, we investigated, at both the gene and protein levels, the expression of CPT1B in CTR, OA, and OP muscle tissue and myoblasts. In muscle tissue, an up-regulation of *CPT1B* in OA patients was observed, compared with CTR and OP subjects, in whom gene expression was comparable. In agreement, the number of Cpt1b-positive muscle fibers resulted also higher in OA muscle tissue compared with both CTR subjects and OP patients, while no significant difference was observed between the percentage of positive fibers between CTR and OP subjects. Further differences were observed instead by analyzing gene and protein expression of CPT1B in the myoblasts of the three patient groups. Indeed, gene expression analysis showed, again, an up-regulation of *CPT1B* in OA myoblasts compared with both CTR and OP cells. In addition, gene down-regulation was observed in OP myoblasts, compared with both OA patients and CTR subjects. This result was confirmed in the subsequent qualitative protein expression analysis conducted through immunofluorescence, which showed that the expression of Cpt1b was higher in OA myoblasts, compared with CTR and OP myoblasts, and lower in OP cells. These results could be explained by the fact that we focused our attention on a specific cell type, i.e., myoblasts, precursor cells of myotubes, and mature muscle fibers. In muscle tissue, instead, results mainly derived from the mature tissue, which includes additional components, such as connective and adipose tissues, as well as the vascular network and the inflammatory component. The presence of these further elements may influence CPT1B expression. In addition to gene and protein expression, we next investigated the enzyme activity levels of Cpt1b in the tissue of the three groups of patients. The results obtained were in line with previous findings; in fact, the activity of Cpt1b resulted higher in OA patients, compared to CTR and OP subjects, among whom it was instead comparable.

Numerous studies in the literature investigated the expression of proteins belonging to the Cpt1 family in the context of oxidative stress, although there are no studies investigating Cpt1b involvement. Indeed, Ding et al. reported how, in an in vitro model of Alzheimer’s disease, the brain protein Cpt1c appears to relieve oxidative stress, in primary hippocampal neurons exposed to β-Amyloid peptide fragment. In this model also, Cpt1c over-expression resulted in improved cell viability, decreased apoptosis, and the decreased expression of pathological markers [27]. In contrast, in Jiang and colleagues’ study, in a mouse model of osteoarthritis, the pharmacological inhibition of Cpt1a appears to alleviate oxidative damage and chondrocyte senescence by regulating mitochondrial dysfunction and mitophagy [28]. Further evidence that there is a close relationship between oxidative stress levels and the functionality of proteins belonging to the Cpt1 family comes from the work of Dong and colleagues investigating the benefits of treatment with the antioxidant Ginsenoside Rb1 on muscle stem cells. In this study, it was observed that this substance alleviates oxidative stress by improving mitochondrial function: specifically, this compound appears to significantly restore the H_2_O_2_-induced loss of total ATP in C2C12 cells, resulting in the up-regulation of genes related to lipid metabolism, including *CPT1A* and *CPT1B* [29]. Therefore, to further investigate the potential involvement of Cpt1b in modulating oxidative stress, we first quantified intracellular ROS levels and then analyzed *NOX4* expression, a major enzyme involved in ROS production. These investigations were conducted on myoblasts, since more differences could be appreciated in cell cultures than those found in mature tissue.

ROS levels resulted higher in OP myoblasts compared with both CTR and OA cells. This result is in line with what has been reported in the literature: high ROS levels are associated with a state of muscle atrophy, which was indeed observed in OP muscle, characterized by a lower percentage of fast myosin-positive fibers and a reduced diameter of all muscle fibers compared to healthy and OA muscle tissue [30]. In contrast, ROS levels resulted lower in OA myoblasts, compared to both CTR and OP myoblasts. Contextually, the up-regulation of *NOX4* was observed in OA myoblasts, both compared to CTR and OP cells, whereas gene expression was found to be down-regulated in OP myoblasts. These results highlight another relevant role played by Nox4, which was, in fact, reported in the work of Youm and colleagues. Experiments conducted on C2C12 *NOX4*-Knockout cells showed that inhibition of this protein results in a lower rate of myoblast fusion, highlighting its important role in contributing to skeletal muscle regeneration and growth [31]. Therefore, the state of muscle atrophy observed in the OP experimental group could be due not only to elevated levels of oxidative stress but also to reduced *NOX4* expression, which would impair the fusion of myoblasts into myotubes and the subsequent generation of mature muscle fibers. In OA patients, on the other hand, the positive role of Nox4 might be predominant, allowing these patients to have muscle tissue characterized by a quality comparable to that of a healthy subject. According to the literature reports also, Nox4 appears to be involved in the process of glucose and fatty acid oxidation; this finding, therefore, seems to suggest a potential close correlation between the expression of this enzyme and the expression and/or activity of CPT1B [32].

Since Cpt1b is localized in the outer mitochondrial membrane, we next wanted to investigate the expression and potential role of Bnip3 in the muscle pathophysiology of osteoarthritis and osteoporosis. Indeed, it is noteworthy that Bnip3 represents a key regulator of mitophagic flux, the proper functioning of which enables myogenic differentiation and the process of myogenesis; indeed, mitophagy represents a cytoprotective mechanism to remove superfluous or dysfunctional mitochondria and thus maintain intracellular homeostasis [33]. We, therefore, qualitatively investigated the expression of Bnip3 in CTR, OA, and OP myoblasts by immunofluorescence. This investigation allowed us to appreciate numerous differences in expression and localization among myoblasts isolated from the muscle of the three groups of patients. Specifically, in CTR myoblasts, the expression of Bnip3 was found to be widespread in the cytoplasm and also higher than that observed in OA and OP cells. Such localization suggests a pro-myogenic and anti-apoptotic role of Bnip3. In contrast, in OA myoblasts, Bnip3 appeared to be aggregated in numerous and distinct spots (puncta), likely autophagosomes, suggesting its greater involvement, in this type of patient, in the mitophagic process. Finally, in OP myoblasts, a lower expression of Bnip3 was observed, compared with both CTR and OA myoblasts but localized purely in the perinuclear zone, suggesting in this case a greater involvement of the protein in the process of myoblast apoptosis, thereby compromising the generation of myotubes and new mature muscle fibers [34]. Finally, given the nature of the previous analyses, we conducted an ultrastructural analysis of the muscle tissue of the three patient groups to more closely assess the quality of muscle fibers and, more specifically, the qualitative state of mitochondria. First, in the muscle fibers of CTR subjects, well-organized sarcomeres were observed, as well as in the muscle of OA patients, whereas the sarcomeres of OP muscle do not appear well-defined. In addition, numerous glycogen granules are distributed in the cytoplasm of the muscle fibers of CTR and OA patients in contrast to the OP group in which they are visible in smaller quantities. Finally, this investigation showed that the mitochondrial qualitative status of CTR and OA subjects was comparable: in fact, in these two groups, mitochondria were found to have a regular shape and well-defined mitochondrial cristae. In contrast, the muscle of OP patients was found to have giant, irregularly shaped mitochondria with disorganized mitochondrial cristae that had lost their integrity.

This study has two main limitations: further in vitro functional studies are needed to have confirmation of the possible direct correlation between CPT1B expression and/or activity and oxidative stress. In addition, the study of other markers would be useful to better characterize mitophagy and energy metabolism in this type of patients.

## 5. Conclusions

Cpt1b represents an important enzyme involved in mitochondrial energy metabolism, which appears to be highly activated in the muscle of OA patients and down-regulated in OP myoblasts. In addition to joint degeneration, osteoarthritis is characterized by a strong inflammatory state, resulting in increased levels of oxidative stress and cellular damage. The up-regulation of Cpt1b in OA muscle could therefore play a protective role against the insults to which the musculoskeletal system of an OA patient is constantly subjected. The up-regulation of Cpt1b could also trigger the initiation of protective mechanisms, which therefore allow the muscle of an OA patient to maintain a qualitative state comparable to that of a healthy subject, preserving good muscle performance in healthy aging. In OP patients, on the other hand, the percentage of Cpt1b-positive fibers and gene expression in myoblasts was lower, while activity was maintained at a level comparable to that of a healthy subject. Such down-regulation could impair not only muscle energy metabolism but also reparative mechanisms against external insults, and this could, therefore, result in the higher levels of oxidative stress present and the characteristic state of muscle atrophy observed. In addition, the impairment of the mitophagic pathway was observed in OP patients, which could further worsen the qualitative state of the muscle fiber, leading to an unhealthy aging. The results of the present study extend the current knowledge of muscle pathophysiology in the two diseases, suggesting that Cpt1b may represent a new potential therapeutic target for the treatment of muscle atrophy that generally accompanies the onset of osteoarthritis and osteoporosis in elderly people.

## Figures and Tables

**Table 1:** Clinical, histological, and biochemical characteristics of OP, OA patients and CTR subjects.

Characteristics	CTR (*n* = 5)	OA (*n* = 10)	OP (*n* = 10)	*p* Value
Age (years)	30.0 ± 6.9	66.6 ± 6.2	64.3 ± 4.8	CTR vs. OA: *** *p* < 0.001 CTR vs. OP: *** *p* < 0.001 OA vs. OP: ns
BMI (kg/cm^2^)	22.6 ± 3.4	25.7 ± 4.8	25.6 ± 4.2	ns
BMD total femur (g/cm^2^)	1.0 ± 2.4	1.2 ± 0.1	0.7 ± 0.0	CTR vs. OA: ns CTR vs. OP: *** *p* < 0.001 OA vs. OP: *** *p* < 0.001
*t*-score total femur	0.0 ± 0.5	1.0 ± 1.0	−2.4 ± 0.2	CTR vs. OA: ns CTR vs. OP: *** *p* < 0.001 OA vs. OP: *** *p* < 0.001
BMD femoral neck (g/cm^2^)	1.1 ± 3.4	1.0 ± 0.1	0.6 ± 0.0	CTR vs. OA: ns CTR vs. OP: *** *p* < 0.001 OA vs. OP: *** *p* < 0.001
*t*-score femoral neck	0.1 ± 0.3	0.0 ± 0.6	−2.8 ± 0.3	CTR vs. OA: ns CTR vs. OP: *** *p* < 0.001 OA vs. OP: *** *p* < 0.001
BMD lumbar vertebrae L1–L4 (g/cm^2^)	1.2 ± 1.1	1.4 ± 0.2	1.1 ± 0.2	CTR vs. OA: ns CTR vs. OP: *** *p* < 0.001 OA vs. OP: *** *p* < 0.001
*t*-score lumbar vertebrae L1–L4	−0.2 ± 0.2	1.6 ± 1.8	−0.8 ± 1.5	CTR vs. OA: ns CTR vs. OP: *** *p* < 0.001 OA vs. OP: *** *p* < 0.001
Muscle fibers’ diameter (µm)	89.4 ± 6.2	84.1 ± 6.6	70.0 ± 1.9	CTR vs. OA: ns CTR vs. OP: * *p* < 0.05 OA vs. OP: ns
Type II muscle fibers (%)	56.3 ± 5.8	43.7 ± 3.2	20.0 ± 14	CTR vs. OA: ns CTR vs. OP: * *p* < 0.05 OA vs. OP: ns
Ca (mg/dL)	8.2 ± 0.6	8.4 ± 0.5	8.3 ± 0.3	ns
25-(OH)-VitD (ng/mL)	22.5 ± 6.1	19.9 ± 10.7	15.5 ± 8.0	ns
PTH (pg/mL)	66.2 ± 34.6	80.7 ± 38.5	84.2 ± 49.0	ns

BMI, body mass index; BMD, bone mineral density; PTH, parathyroid hormone; 25-(OH)-VitD, 25-hydroxyvitamin D.

**Table 2:** The qRT-PCR primer sequences.

Gene		Sequence (5′–3′)
*CPT1B*	Reverse Forward	TCTCGCCTGCAATCATGTAG ATTCTCATCGCTTTGGAAGG
*NOX4*	Reverse Forward	AGAGGAACACGACAATCAGCCTTAG CTCAGCGGAATCAATCAGCTGTG
*GAPDH*	Reverse Forward	GTCTTCTGGGTGGCAGTGAT GATCATCAGCAATGCCTCCTG

## Data Availability

Raw data are made available upon request to the authors.
